# Luseogliflozin, a SGLT2 Inhibitor, Does Not Affect Glucose Uptake Kinetics in Renal Proximal Tubules of Live Mice

**DOI:** 10.3390/ijms22158169

**Published:** 2021-07-29

**Authors:** Anqi Zhang, Daisuke Nakano, Wararat Kittikulsuth, Yuka Yamashita, Akira Nishiyama

**Affiliations:** Department of Pharmacology, Kagawa University, Takamatsu 760-8521, Kagawa, Japan; s18d715@stu.kagawa-u.ac.jp (A.Z.); wararat.kittikulsuth@kagawa-u.ac.jp (W.K.); s18m101@stu.kagawa-u.ac.jp (Y.Y.); nishiyama.akira@kagawa-u.ac.jp (A.N.)

**Keywords:** glucose handling, proximal tubules, intravital imaging

## Abstract

Proximal tubules (PTs) take up most of the glucose in the glomerular filtrate and return it to peritubular capillary blood. Sodium-glucose cotransporter 2 (SGLT2) at the apical membrane takes up glucose into the cell. Glucose then flows across the cells and is transported to the interstitium via glucose transporter 2 (GLUT2) at the basolateral membrane. However, glucose transport under SGLT2 inhibition remains poorly understood. In this study, we evaluated the dynamics of a fluorescent glucose analog, 2-NBDG, in the PTs of live mice treated with or without the SGLT2 inhibitor, luseogliflozin. We employed real-time multiphoton microscopy, in which insulin enhanced 2-NBDG uptake in skeletal muscle. Influx and efflux of 2-NBDG in PT cells were compared under hypo-, normo-, and hyperglycemic conditions. Luseogliflozin did not exert significant effects on glucose influx parameters under any level of blood glucose. Our results suggest that blood glucose level per se does not alter glucose influx or efflux kinetics in PTs. In conclusion, neither SGLT2 inhibition nor blood glucose level affect glucose uptake kinetics in PTs. The former was because of glucose influx through basolateral GLUT2, which is an established bidirectional transporter.

## 1. Introduction

In modern life, owing to improvements in living conditions and enrichment of food types, excessive energy intake has created new challenges that threaten the health of individuals. These challenges include diabetes. Accordingly, treatment and prevention of diabetes are becoming increasingly important. Inhibitors of sodium-glucose cotransporter 2 (SGLT2) act via a mechanism that is different from that of other antihyperglycemic drugs for reducing blood glucose levels. Furthermore, compared with other antihyperglycemic drugs, there is increasing evidence that SGLT2 inhibitors reduce the risk of cardiorenal-vascular events [1,2]. Therefore, there is high demand for knowledge on how SGLT2 inhibitors confer favorable effects.

Large amounts of glucose are reabsorbed in the convoluted segment of the proximal tubules. SGLT2, expressed on brush border membranes, transports sodium and glucose from the tubular lumen into the cells. SGLT2 inhibitors reduce glucose uptake in proximal tubule cells, which increases glucose excretion in urine, thereby reducing blood glucose levels under hyperglycemic conditions. It is believed that glucose taken up through SGLT2 flows across the cells and is transported to the interstitium. Glucose transporter 2 (GLUT2), expressed in the basement membrane, transports glucose from the intracellular space to interstitial fluid.

GLUT2 is an important glucose transporter and is widely distributed in various organs and tissues, such as the liver, intestine, kidney, pancreatic islet β-cells, and central nervous system [3]. Additionally, GLUT2 facilitates bidirectional glucose transport [4]. When the blood glucose level is high, blood glucose passes through GLUT2 expressed in the liver and is stored as glycogen. When the blood glucose level is low, the liver promotes glycogen decomposition to glucose and provides glucose to the blood through GLUT2 [5]. In the kidney, GLUT2 is coexpressed with SGLT2 [6]. In our previous study using intravital imaging at a subcellular spatial resolution, we found that GLUT2 downregulation dramatically reduced glucose uptake into the proximal tubules of mice treated with luseogliflozin, a SGLT2 inhibitor [7]. This observation suggested the potential for glucose uptake by GLUT2 in the kidney under the influence of SGLT2 inhibitors. This prompted our interest in determining whether SGLT2 inhibition using luseogliflozin affects glucose uptake dynamics in the proximal tubule cells of live mice. We also evaluated proximal tubule glucose uptake dynamics with and without luseogliflozin under different levels of blood glucose. We used fluorescent glucose analogs 2-deoxy-2-[(7-nitro-2,1,3-benzoxadiazol-4-yl) amino]-D-glucose (2-NBDG) in vivo and 2-deoxyglucose in vitro to visualize glucose dynamics.

## 2. Results

### 2.1. In Vivo Analysis of Glucose Dynamics in Skeletal Muscle Using Multiphoton Imaging

**Figure 1:**
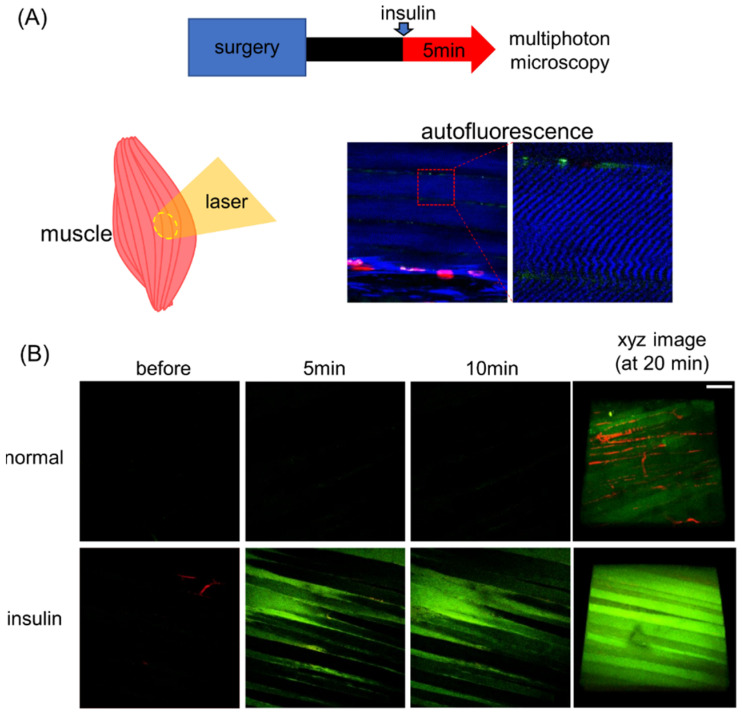
Intravital imaging for skeletal muscle glucose uptake. (**A**) Protocol for the skeletal muscle experiment. Insulin was injected 5 min before injection of the fluorescent glucose analog, 2-deoxy-2-[(7-nitro-2,1,3-benzoxadiazol-4-yl)amino]-D-glucose (2-NBDG). We observed skeletal muscle of the medial thigh of mice, and the focal plane was selected based on autofluorescence from muscle fibers (blue). (**B**) Representative in vivo images of 2-NBDG (green) uptake in mouse skeletal muscle before and after 2-NBDG injection (*n* = 4). Scale bar = 80 µm.

We first confirmed the reliability of our in vivo imaging system by measuring the insulin-accelerated 2-NBDG uptake in skeletal muscle. Insulin was intravenously injected 5 min before 2-NBDG administration. The focal plane was selected based on the autofluorescence of skeletal muscle, which was excited at a wavelength of 860 nm (Figure 1A). As shown in Figure 1B, insulin time-dependently increased 2-NBDG-derived fluorescence (green) in skeletal muscle (Figure 1B). The result supports the reliability of our multiphoton imaging system for visualization of in vivo glucose dynamics by using the fluorescent glucose analog, 2-NBDG.

### 2.2. Analysis of Glucose Dynamics in Proximal Tubules of Live Animals

**Figure 2:**
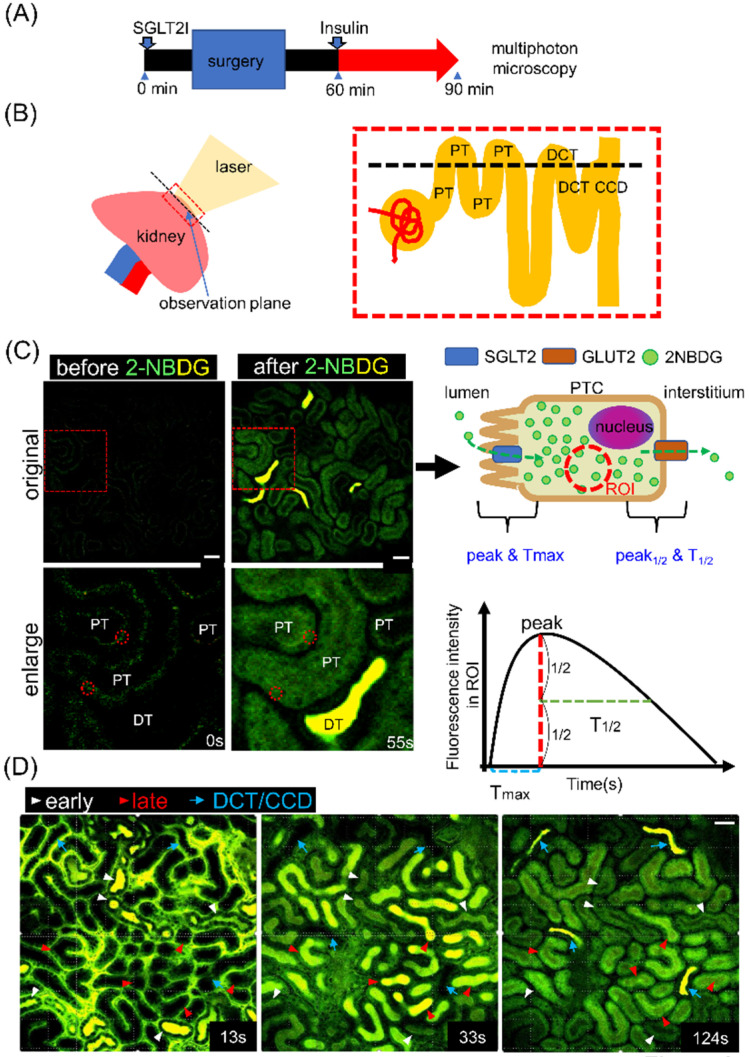
Intravital imaging for proximal tubule glucose uptake. (**A**) Protocol for the kidney experiment. Either vehicle or luseogliflozin was injected 90 min before 2-NBDG injection. Insulin was injected 30 min before injecting 2-NBDG. (**B**) The microscopic observation plane was at the superficial level of the kidney cortex that includes proximal tubules (PTs) near the glomerulus, PTs relatively far from the glomerulus, distal convoluted tubules (DCT), and collecting ducts (CCD). (**C**) In vivo image before and after 2-NBDG injection. Regions of interest (ROI) were set in the cytosol of PT cells (PTCs) (red dotted circles), and changes in fluorescence intensity were measured. 2-NBDG at a low level was depicted as green, while a saturated level of 2-NBDG was depicted as yellow (green and red). (**D**) In vivo images at different time points after 2-NBDG injection (13, 33, and 124 s). The image on the left (13 s) shows 2-NBDG in the lumen of early segments of PT and peritubular capillaries (white arrows). The image at the center (33 s) shows 2-NBDG in the lumen of late segments of PTs (red arrows) and in the cytosol of early segments. The image on the right (124 s) shows 2-NBDG in the lumen of DCT or collecting ducts (CDs) (blue arrow) and in the cytosol of PTC. The image before 2-NBDG injection was as dark as the image provided in C. Scale bar = 40 µm.

Next, we evaluated 2-NBDG uptake in the kidneys of live mice. We injected luseogliflozin intraperitoneally 90 min before 2-NBDG administration [8]. In some mice, insulin was injected 30 min before 2-NBDG administration (Figure 2A). Superficial areas of the kidney at a 20–40-μm depth were visualized using two-photon microscopy. The focal plane contained convoluted segments of proximal tubules, distal convoluted tubules, and cortical collecting duct (Figure 2B), and proximal tubules could be identified based on autofluorescence (Figure 2C). 2-NBDG is depicted as green to yellow (if saturated) throughout the figures. We set a region of interest in the cytosol of individual cells and measured time-dependent changes in fluorescence intensity (Figure 2C). Peak (maximum level), Tmax (time to achieve the peak level), and T1/2 (time to halve the fluorescence level from the peak) were analyzed (Figure 2C). A portion of 2-NBDG was not taken up and flowed into the distal nephron. This enabled us to identify earlier and later segments of the proximal tubule based on the time 2-NBDG appeared in the tubular lumen (Figure 2D).

### 2.3. Effect of Luseogliflozin on Glucose Dynamics in Proximal Tubules

**Figure 3:**
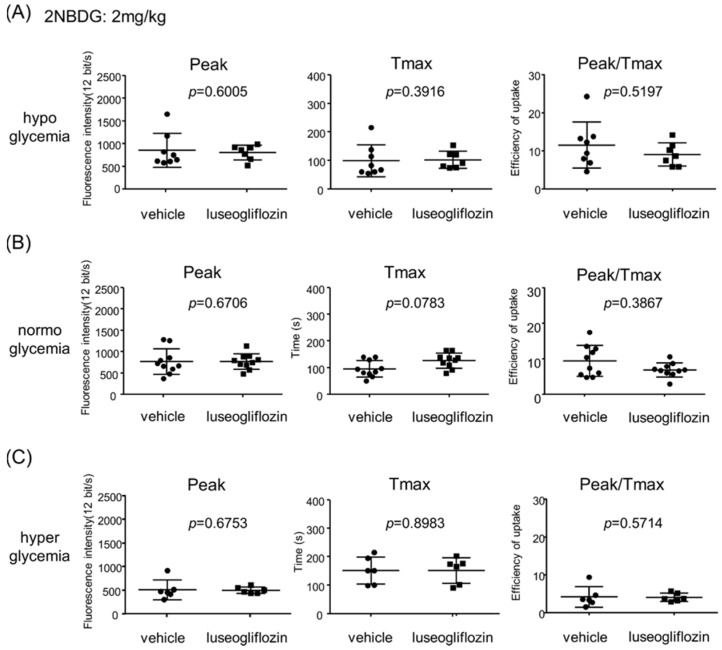
Effects of luseogliflozin on 2-NBDG (2 mg/kg) dynamics in early segments of proximal tubules. Peak, Tmax and peak/Tmax were compared under (**A**) hypoglycemic, (**B**) normoglycemic, and (**C**) hyperglycemic conditions. Each dot represents an average value from five proximal tubule cells in each mouse (*n* = 7–10).

Blood glucose level in mice was measured just before 2-NBDG injection (Table 1). Luseogliflozin did not significantly affect blood glucose level in the either hypo- or normoglycemia groups (Table 1). In the streptozotocin (STZ)-induced hyperglycemia group, luseogliflozin decreased blood glucose levels, indicating inhibition of proximal tubule glucose uptake at the apical side. After administering 2-NBDG, peak, Tmax, and peak/Tmax were analyzed separately in the three groups according to blood glucose level (earlier segments: Figure 3 and Supplementary Figures S1–S3; later segments: Supplementary Figures S4 and S5). Luseogliflozin did not significantly affect these parameters in any of the groups, suggesting the existence of an SGLT2-independent glucose transport pathway.

### 2.4. Effects of Blood Glucose Level on Glucose Dynamics in Proximal Tubules

**Figure 4:**
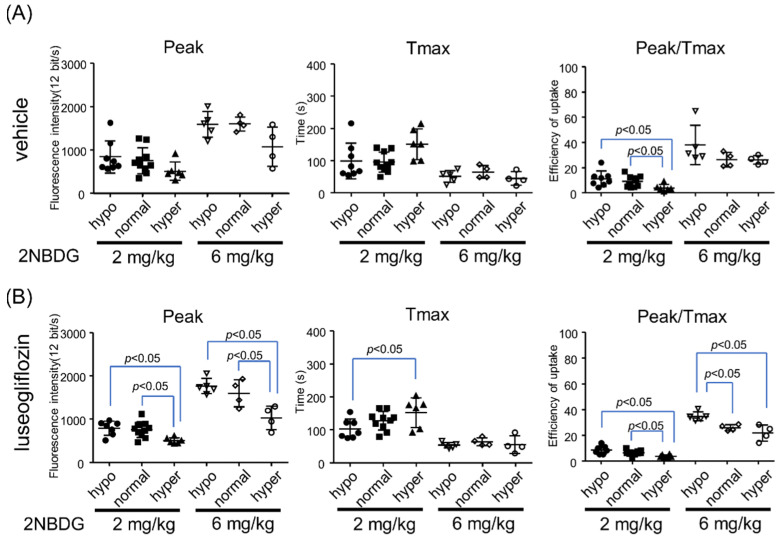
Effects of blood glucose level on 2-NBDG (2 or 6 mg/kg) dynamics in early segments of proximal tubules. Peak, Tmax, and peak/Tmax were compared in (**A**) vehicle- and (**B**) luseogliflozin-treated groups. Hypo: hypoglycemia, normal: normoglycemia, hyper: hyperglycemia. Each dot represents an average value from five proximal tubule cells in each mouse (*n* = 7–10 for the 2 mg/kg group and *n* = 4 for the 6 mg/kg group).

There was no significant difference in peak or Tmax between the hypo- and normoglycemia groups, whether vehicle- or luseogliflozin-treated (Figure 4 and Supplementary Figure S6). In the hyperglycemia groups, peak and peak/Tmax were lower compared with the other groups, possibly because of competition with native glucose at the transporters. Indeed, an increase in the dose of 2-NBDG from 2 to 6 mg/kg resulted in a gain of fluorescence intensity and elimination of the difference in peak/Tmax between the normo- and hyperglycemia groups. A higher dose of 2-NBDG induced saturation of fluorescence level. Importantly, the trend was similar between the vehicle- and luseogliflozin-treated groups.

### 2.5. Effect of Blood Glucose Level on Basolateral Glucose Transport in Proximal Tubules

**Figure 5:**
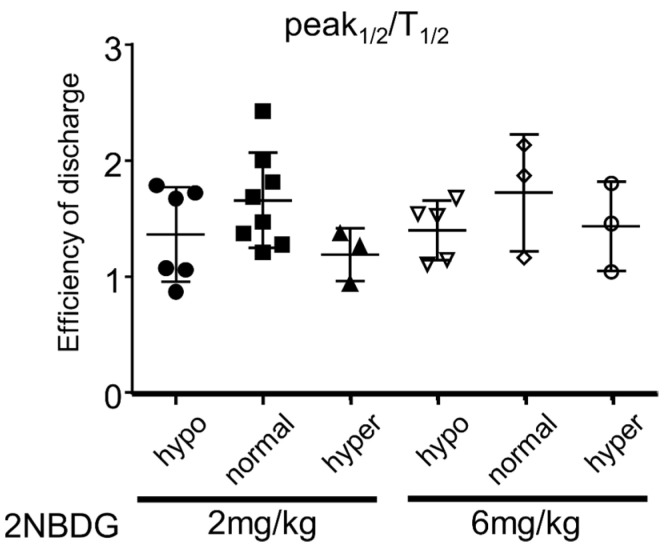
Effects of blood glucose level on 2-NBDG (2 or 6 mg/kg) transport from cytosol to interstitium in early segments of proximal tubules. P1/2/T1/2 was compared between groups. Hypo: hypoglycemia, normal: normoglycemia, hyper: hyperglycemia. Each dot represents an average value from five proximal tubule cells in each mouse (*n* = 3–8 for the 2 mg/kg group and *n* = 3–5 for the 6 mg/kg group).

Physiologically, the glucose taken up through apical membrane SGLT2 flows across the cells and is transported to the interstitium via GLUT2 at the basolateral membrane. Basolateral transport of intracellular glucose to the interstitial fluid was analyzed by dividing half of the peak fluorescence intensity by T1/2. This analysis was performed only in the vehicle-treated group because luseogliflozin treatment markedly prolonged T1/2. Blood glucose level did not significantly affect basolateral glucose transport in proximal tubules (Figure 5).

**Figure 6:**
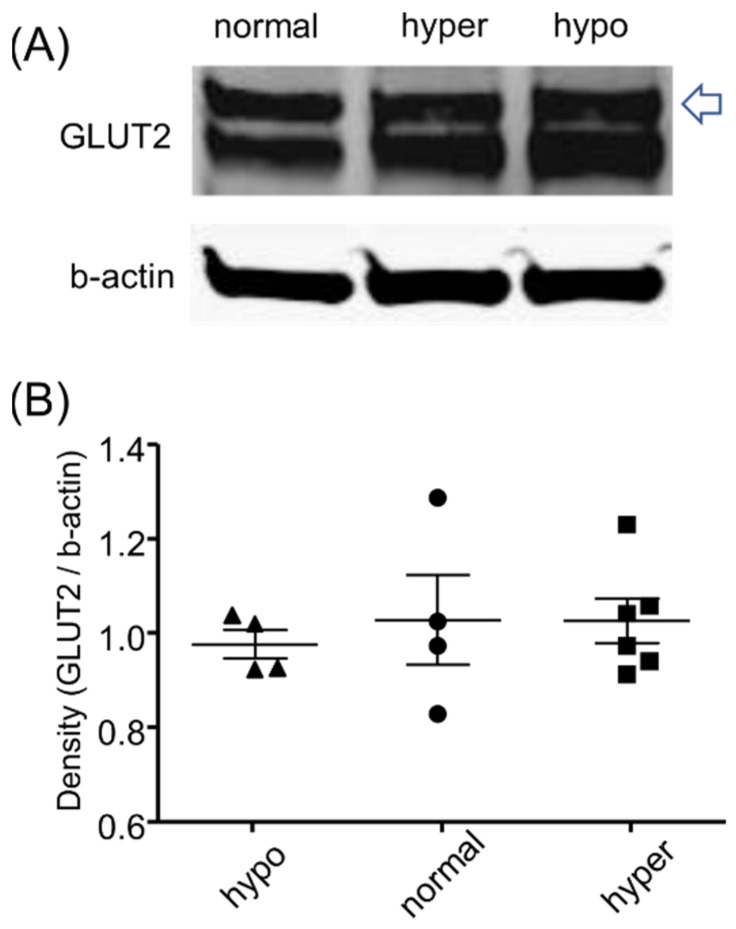
GLUT2 expression in renal cortical tissues assessed by Western blot. (**A**) Representative blots and (**B**) quantitative data are shown (*n* = 4–6). There are two bands for GLUT2; one is membrane-bound form and another is cytosolic form. The bands that are indicated by an arrow, which are detectable mainly in the cell membrane (glycosylated), were analyzed. Hypo: hypoglycemia, normal: normoglycemia, hyper: hyperglycemia.

Changes in GLUT2 level in hypo- and hyperglycemia could affect the glucose transport efficacy at the basolateral membrane of proximal tubules. Because we did not find any significant difference in basolateral glucose transport among the groups, we hypothesized that GLUT2 level in the kidney was similar in the current experimental setting. Thus, we examined GLUT2 protein levels in the three groups of mice with different blood glucose levels. As expected, GLUT2 protein levels were similar in all groups (Figure 6 and Supplementary Figure S7).

### 2.6. Effects of Luseogliflozin on Glucose Uptake in Cultured Proximal Tubule Cells

The in vivo analyses described above indicate that SGLT2 inhibition by luseogliflozin did not change glucose dynamics in proximal tubules. We further confirmed that luseogliflozin does not interfere with GLUT-dependent glucose uptake. Mouse proximal tubule cells (mProx24 cells) cultured in standard DMEM without modification did not demonstrate a sodium-dependent increase in glucose uptake (Supplementary Figure S3A), indicating that the cells do not express functional SGLT2. Glucose uptake in these cells was time-dependently increased as measured using 2-deoxyglucose (Supplementary Figure S3B). Uptake of 2-deoxyglucose was suppressed when it was added to cells cultured in medium containing a relatively higher amount of glucose, such as 25 mM (high) and 17.5 mM (medium), compared with 5 mM (low) glucose-containing medium (Supplementary Figure S3B), indicating competitive inhibition. Preincubation of cells with either low, medium, or high-glucose-containing medium did not affect subsequent 2-deoxyglucose uptake in cells in glucose-free medium (Supplementary Figure 3C), suggesting potential translocation of cytosolic GLUT to the membrane under high glucose was not significant. Importantly, luseogliflozin did not affect 2-deoxyglucose uptake in any of the above experiments. Additionally, incubation of 2-deoxyglucose with luseogliflozin did not affect the uptake (Supplementary Figure S3D). Taken together, luseogliflozin did not affect glucose uptake through GLUT.

## 3. Discussion

SGLT2 inhibitors, a novel class of drug used for treatment of diabetes, increase urinary glucose excretion by inhibiting the major glucose transporter in the kidney, SGLT2, thereby controlling the blood glucose level [1]. SGLT2 inhibitors block glucose uptake at the basolateral membrane of proximal tubules and, in turn, affect urinary glucose excretion. However, glucose dynamics from the lumen to tubular cells and from tubular cells to the interstitium have not been sufficiently examined. In our study, we obtained no evidence showing luseogliflozin alters glucose uptake kinetics in the proximal tubules at multiple levels of extracellular glucose. GLUT2 presumably plays a major role in proximal tubule glucose uptake when SGLT2 is inhibited, and our data suggest the glucose uptake kinetics regulated by GLUT2 are similar to those by SGLT2 in renal proximal tubules of live mice.

It was initially expected that SGLT2 inhibition would slow glucose dynamics in proximal tubules. However, it was unaffected. This led us to speculate that GLUT2, a facilitating transporter expressed in the basement membrane in the liver [5], transferred 2-NBDG from interstitial fluid to proximal tubules. We previously showed that SGLT2 inhibitor alone had a minor effect on glucose uptake, at one specific time point, in the proximal tubules of the nondiabetic kidney and that cellular injury accompanied by reduced GLUT2 expression dramatically halted glucose uptake [7]. In this study, we found that luseogliflozin did not affect time-dependent changes in glucose uptake in mice with either hypo-, normo-, or hyperglycemia, indicating that glucose transport through SGLT2 and GLUT2 was similar and unaffected by extracellular glucose levels. A limitation of our study was the duration of hypo- (<1 h) and hyperglycemia (1 week). We did not use longer experimental periods because chronic exposure to low or high blood glucose can affect transporter expression levels. As mentioned above, we demonstrated that a decreased level of GLUT2 critically affects glucose uptake. Accordingly, we did not chronically treat mice with luseogliflozin because it is well established that it protects the kidneys [7,9], and its potential effects on the expression of transporters can affect experimental results.

It has been shown that there are differences in cellular features and metabolism between early and late segments of proximal convoluted tubules [10,11,12]. Therefore, we separately analyzed 2-NBDG dynamics in early and late segments. According to our observations, use of luseogliflozin did not significantly change parameters related to glucose dynamics in either early or late segments, regardless of levels of blood glucose.

One of our focus areas was the effect of blood glucose level on glucose kinetics in proximal tubule cells. The hyperglycemia group demonstrated a lower peak/Tmax than the other two groups. This may have resulted from competition between native glucose and 2-NBDG. This should be overcome by constructing dose–response curves using graded doses of 2-NBDG. The dose of 2-NBDG that was three times higher (6 mg/kg) than the original dose (2 mg/kg) resulted in increased fluorescence intensity, indicating that under the condition of hyperglycemia, 2-NBDG uptake by the kidney could afford to be more. Therefore, in the hyperglycemia group, total glucose uptake, either through SGLT2 or GLUT2, was estimated to be greater than in the other groups because of a higher blood glucose level. It should be noted that the primary regulatory factor of SGLT2, sodium concentration, is much higher than the glucose concentration and that the Km value of GLUT2 is over double the physiological plasma glucose concentration [13]. However, further increasing the dose of 2-NBDG, such as to 10 mg/kg, resulted in saturation of the fluorescence level, making it impossible to analyze glucose transport efficiency. Technology that allows for analysis of glucose transport through both apical and basolateral membranes in real time with high-sensitivity quantification is needed.

The original dose of 2-NBDG was 2 mg/kg, which is estimated to be 5 mg/dL in the blood of a 25 g mouse with 1 mL of plasma. Therefore, artificial increases in blood sugar level by injection of 2-NBDG were negligible in the in vivo experiments.

Another limitation was that our experimental design could not account for glucose generated intracellularly via gluconeogenesis. Although we previously reported that inhibition of glucose uptake by SGLT2 siRNA or removal of glucose from medium is sufficient to induce cell phenotypic changes, such as suppression of cell senescence [14] or an increase in angiogenic factor secretion [7], the intracellular glucose level must be taken as the sum of glucose taken up from extracellular spaces and that generated intracellularly. SGLT2 inhibitors reportedly increase expression of genes involved in gluconeogenesis [15,16]. An increase in intracellular glucose production via de novo pathways may attenuate the concentration gradient of glucose between the cytosol and interstitium, thereby counteracting glucose influx through GLUT2. However, to date, there are technical limitations associated with comparing amounts of glucose via gluconeogenesis or glucose uptake.

In vivo multiphoton imaging allows for testing of glucose uptake by the visualization of changes in 2-NBDG uptake in the tissue of live animals. Real-time analysis was performed in skeletal muscle [17,18] but not in the kidney. Skeletal muscle has long been shown to take up glucose from blood via glucose transporter 4 (GLUT4). When insulin levels are elevated, insulin receptors in skeletal muscle produce signals that stimulate the transfer of GLUT4 from the cytoplasm to the cell membrane [19,20]. Glucose uptake in skeletal muscle increases with expression of GLUT4 on the cell membrane, resulting in a reduced blood glucose level [21,22]. Owing to this process, we initially used skeletal muscle to confirm the functionality of our analytical system for examining glucose dynamics. We found that insulin significantly and time-dependently increased skeletal muscle uptake of 2-NBDG, consistent with previous reports [23].

In conclusion, treatment with luseogliflozin did not cause changes in glucose uptake kinetics in the proximal convoluted tubules of the kidney in normal mice. Bidirectional transport of GLUT2 may be the main factor why the proximal convoluted tubule maintains similar glucose uptake kinetics following luseogliflozin treatment.

## 4. Materials and Methods

### 4.1. Materials

Luseogliflozin was provided by Taisho Pharmaceutical Co., Ltd. (Tokyo, Japan). All other chemicals were from Sigma (St. Louis, MO, USA) or Wako unless otherwise specified.

All experimental procedures using animals were performed according to guidelines for the care and use of animals established by Kagawa University.

### 4.2. In Vivo Imaging

Six-week-old male C57BL/6J mice were from Clea Japan (Tokyo, Japan). Mice received either vehicle (4.5% HP-β-CD, intraperitoneal) or luseogliflozin (0.9 mg/kg, intraperitoneal) and were then anesthetized with 1.0–1.5% isoflurane (Mylan, Osaka, Japan). All surgery and animal maintenance during intravital imaging procedures were performed as reported previously [24,25]. After tracheotomy, the jugular vein was cannulated for injection of fluorescent dye and insulin (0.64 U/kg, intravenous). The left kidney of each mouse was exteriorized through a small flank incision and attached to a coverslip. The microscope stage and animals were warmed using a heating pad during all experimental procedures. Intravital multiphoton microscopy was performed using an Olympus FV1000MPE multiphoton confocal fluorescence imaging system, powered by a Chameleon Ultra-II MP laser at 860 nm (Coherent Inc., Santa Clara, CA, USA). The microscope objective was a 25× water immersion lens with a 1.05 numerical aperture. Imaging settings for the microscope (gain and offset for all three channels; blue, green, and red) were fixed throughout the experiments. Two-dimensional time-lapse images were taken 30 µm from the surface of the kidney. Time-lapse images were obtained at a resolution of 512 × 512 pixels with excitation at 860 nm. To visualize glucose uptake in proximal tubules in vivo, we intravenously injected a fluorescent glucose analog, 2-NBDG (2 or 6 mg/kg). The cytosolic fluorescence intensity of 2-NBDG was measured in five different proximal tubular cells in the early and late segments, respectively, for 30 min. Peak, Tmax, and T1/2 values were extracted based on time-dependent changes in fluorescence intensity in each cell. Each mouse was injected with approximately 40–50 µg of 2-NBDG. The estimated plasma 2-NBDG level was around 40–50 mg/L (the hypothesized amount of plasma in a mouse is approximately 1 mL). Therefore, injection of 2-NBDG was unlikely to have caused excess hyperglycemia (glucose + 2-NBDG in mouse blood). For muscle visualization, skeletal muscle on the medial side of the right thigh was surgically cut and exposed and was attached to a coverslip.

Hyperglycemia was induced by STZ injection. C57BL/6J mice were fasted 8 h before STZ injection (50 mg/kg per day, 5 consecutive days). STZ was dissolved in sodium citrate buffer (pH 4.5) just before injection. Blood glucose level was confirmed on Day 5 after the final injection, and experiments were performed on Day 7.

Urinary protein excretion was determined using a commercially available assay kit (microTP-test; Wako, Osaka, Japan).

### 4.3. Western Blot

Whole kidney tissue was homogenized using a Physcotoron (Microtec, Chiba, Japan) in 10% *w/v* buffer (10 mM Tris-HCl, 1 mM EDTA, and 150 mM sucrose, pH 7.4, containing proteinase/phosphatase inhibitor cocktail (#5871; Cell Signaling, Danvers, MA, USA) diluted at 1:100) and centrifuged at 4000× *g* for 120 s. Equal amounts of protein (50 µg) were subjected to SDS-PAGE and transferred to nitrocellulose membranes. After blocking, the membranes were incubated with anti-GLUT-2 antibody (1:1000 dilution; 07-1402-I, Millipore, Billerica, MA, USA).

### 4.4. Cell Culture

Mouse proximal tubule cells (mProx24 cell line) were kindly provided by Dr. Takeshi Sugaya (Timewell Medical Co., Ltd., Tokyo, Japan). Cells were cultured at 37 °C, 5% CO_2_ until 90% confluent, and were then incubated for 24 h before starting experiments with fetal bovine serum (FBS)-free DMEM containing 17.5 mM D-glucose. Cells were incubated in FBS-free DMEM, with glucose levels adjusted to 5, 17.5, or 25 mmol/L, with either vehicle (2-hydroxypropyl-β-cyclodextrin) or luseogliflozin (100 nmol/L, which sufficiently blocks SGLT2 [26]). The in vitro glucose uptake tests were performed using a commercially available kit (2-Deoxyglucose Uptake Measurement kit, Cosmo Bio, Tokyo, Japan) according to the manufacturer’s instructions.

### 4.5. Statistical Analysis

Statistical significance was assessed using one-way ANOVA followed by Tukey’s multiple comparison test to evaluate differences among the multiple groups. Mann–Whitney’s U tests were performed to compare the differences between the 2 groups. All statistical analyses were performed using GraphPad Prism 5 (GraphPad Software Inc., La Jolla, CA, USA), with *p* < 0.05 considered statistically significant. Data are presented as mean ± standard error of the mean.

## Figures and Tables

**Table 1:** Blood glucose levels (mg/dL) before 2-NBDG injection.

	Hypoglycemia	Normoglycemia	Hyperglycemia
Vehicle	51.69 ± 1.90 (*n* = 13)	146.58 ± 6.70 (*n* = 12)	412.42 ± 20.29 (*n* = 12)
Luseogliflozin	53.67 ± 5.13 (*n* = 12)	139.67 ± 7.97 (*n* = 12)	296.75 ± 19.08 * (*n* = 12)

* *p* < 0.05 vs. vehicle.

## Data Availability

All relevant raw data pertaining to the data presented in the manuscript or supplementary figures and tables are available from the authors of the study upon request.

## Supplementary Materials

The following are available online at https://www.mdpi.com/article/10.3390/ijms22158169/s1, Figure S1: Effects of luseogliflozin on 2-NBDG (2 mg/kg) dynamics in early segments of proximal tubules (data from each tubular cell). Figure S2: Effects of luseogliflozin on 2-NBDG (6 mg/kg) dynamics in early segments of proximal tubules (data from each mouse). Figure S3: Effects of luseogliflozin on 2-NBDG (6 mg/kg) dynamics in early segments of proximal tubules (data from each tubular cell).Figure S4: 2-NBDG (2 and 6 mg/kg) dynamics in late segments of proximal tubules (data from each mouse). Figure S5: 2-NBDG (2 and 6 mg/kg) dynamics in late segments of proximal tubules (data from each tubular cell). Figure S6: Effects of blood glucose level on 2-NBDG (2 or 6 mg/kg) dynamics in early segments of proximal tubules (data from each tubular cell). Figure S7: Effects of blood glucose level on 2-NBDG (2 or 6 mg/kg) transport from cytosol to interstitium in early segments of proximal tubules (data from each tubular cell). Figure S8: Uptake of 2-deoxyglucose (2-DG) in cultured proximal tubular cells.
