# Supplementary material for: Luseogliflozin, a SGLT2 Inhibitor, Does Not Affect Glucose Uptake Kinetics in Renal Proximal Tubules of Live Mice
Source: Int J Mol Sci. 2021 Jul 29;22(15):8169. doi: 10.3390/ijms22158169 (PMC8347119; doi:10.3390/ijms22158169)
Supplement: Supplementary file 1 [file ijms-22-08169-s001.zip › ijms-1265619-supplementary.pptx]

## Slide 1
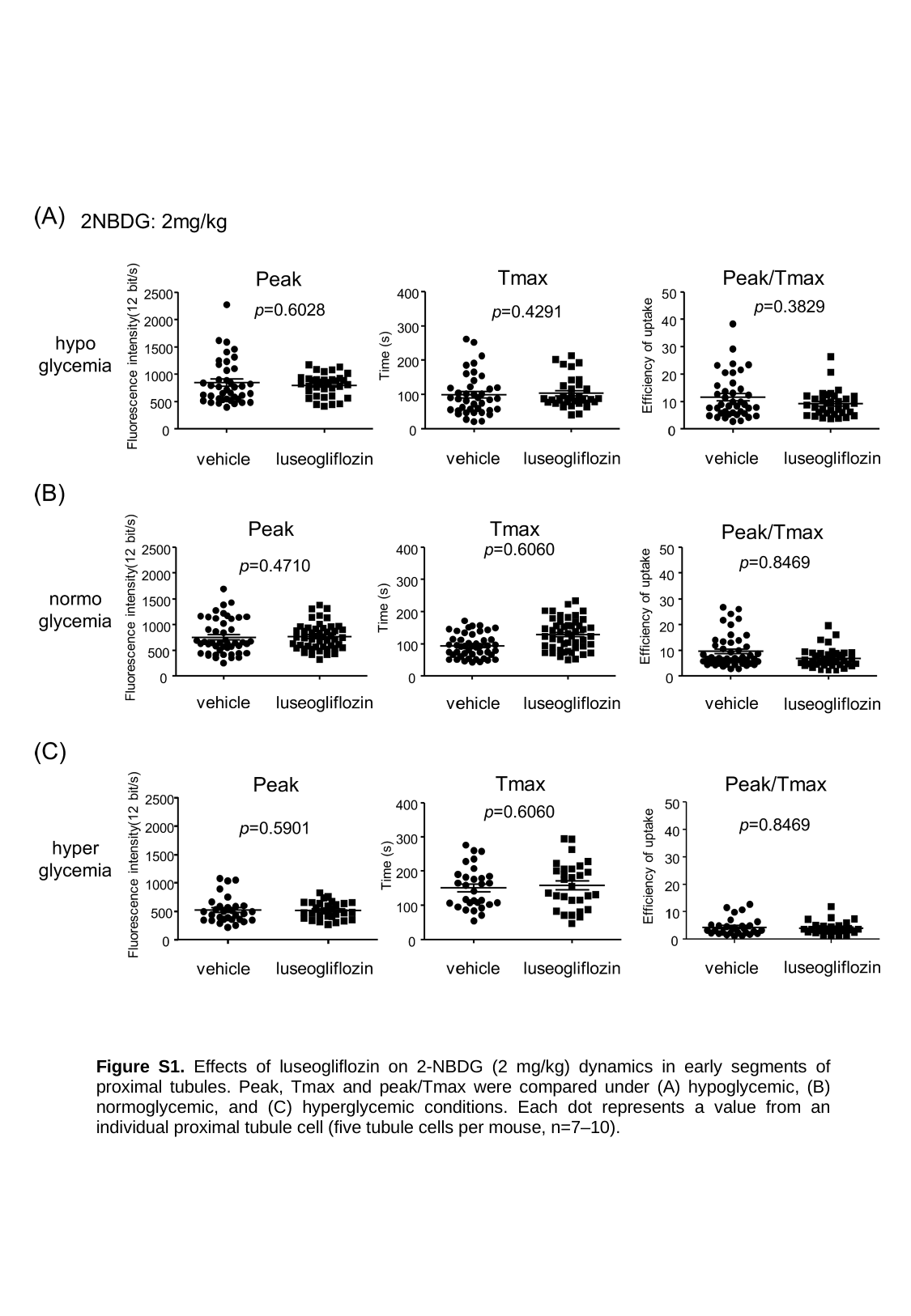

Figure S1. Effects of luseogliflozin on 2-NBDG (2 mg/kg) dynamics in early segments of proximal tubules. Peak, Tmax and peak/Tmax were compared under (A) hypoglycemic, (B) normoglycemic, and (C) hyperglycemic conditions. Each dot represents a value from an individual proximal tubule cell (five tubule cells per mouse, n=7–10).

## Slide 2
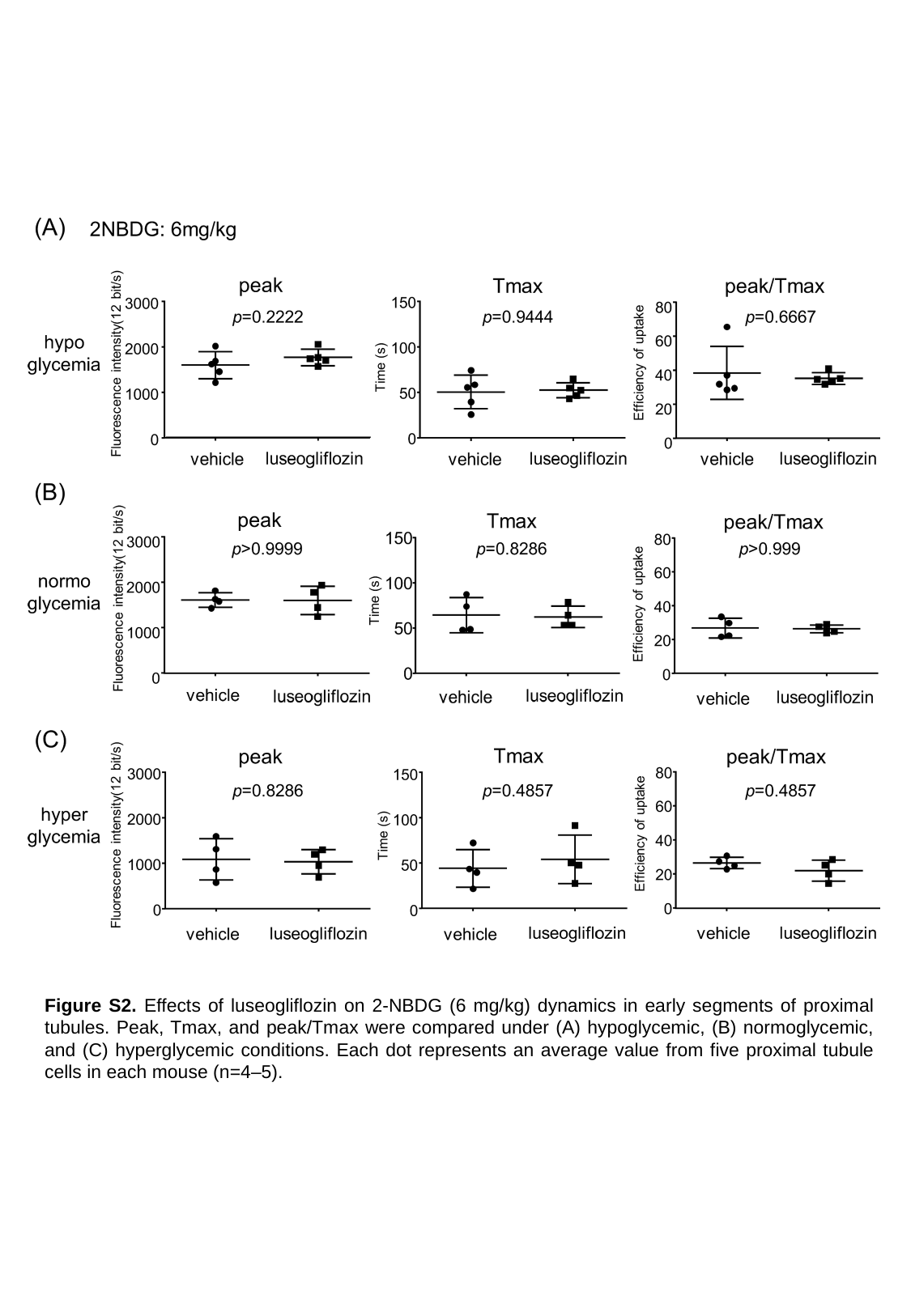

Figure S2. Effects of luseogliflozin on 2-NBDG (6 mg/kg) dynamics in early segments of proximal tubules. Peak, Tmax, and peak/Tmax were compared under (A) hypoglycemic, (B) normoglycemic, and (C) hyperglycemic conditions. Each dot represents an average value from five proximal tubule cells in each mouse (n=4–5).

## Slide 3
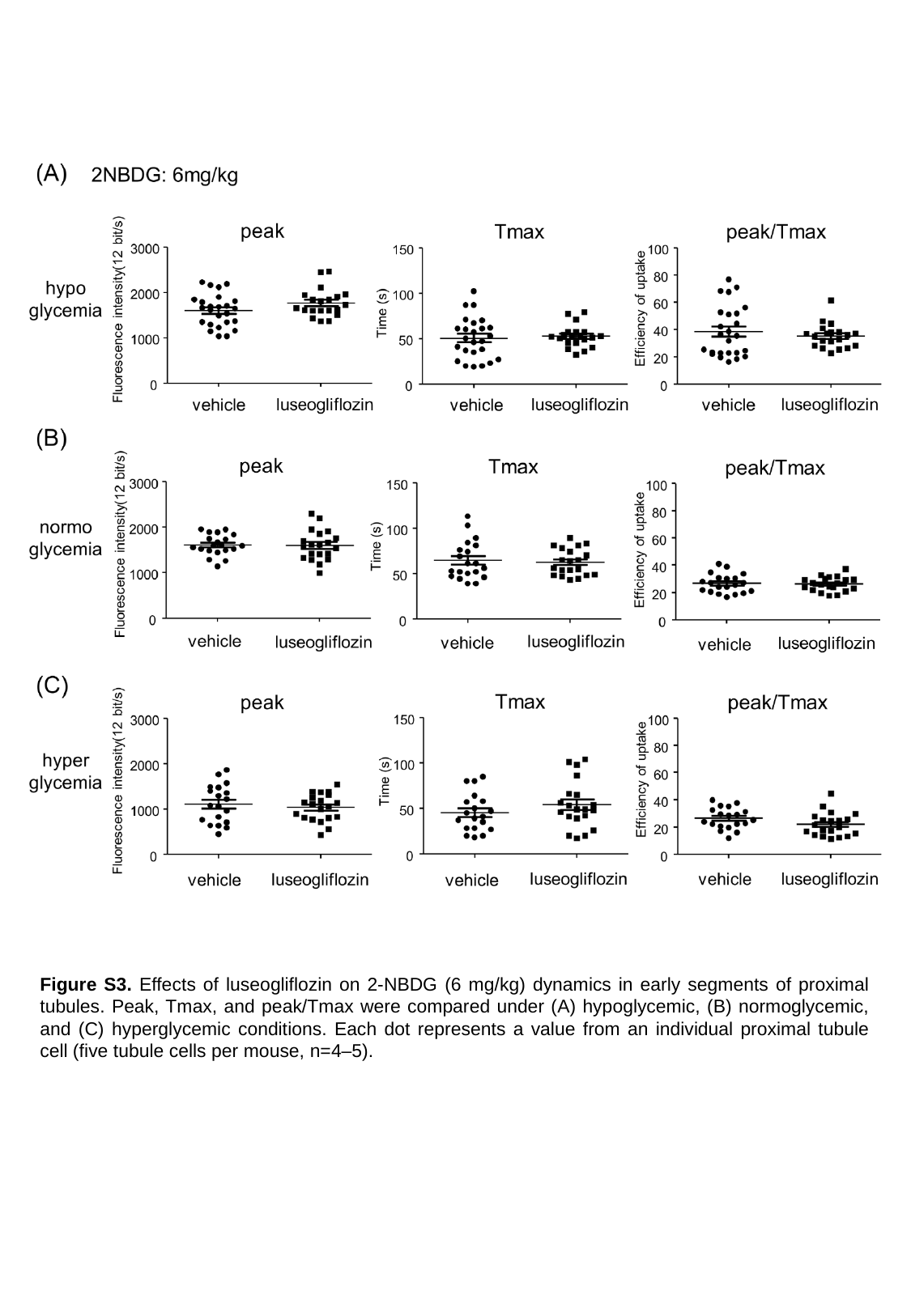

Figure S3. Effects of luseogliflozin on 2-NBDG (6 mg/kg) dynamics in early segments of proximal tubules. Peak, Tmax, and peak/Tmax were compared under (A) hypoglycemic, (B) normoglycemic, and (C) hyperglycemic conditions. Each dot represents a value from an individual proximal tubule cell (five tubule cells per mouse, n=4–5).

## Slide 4
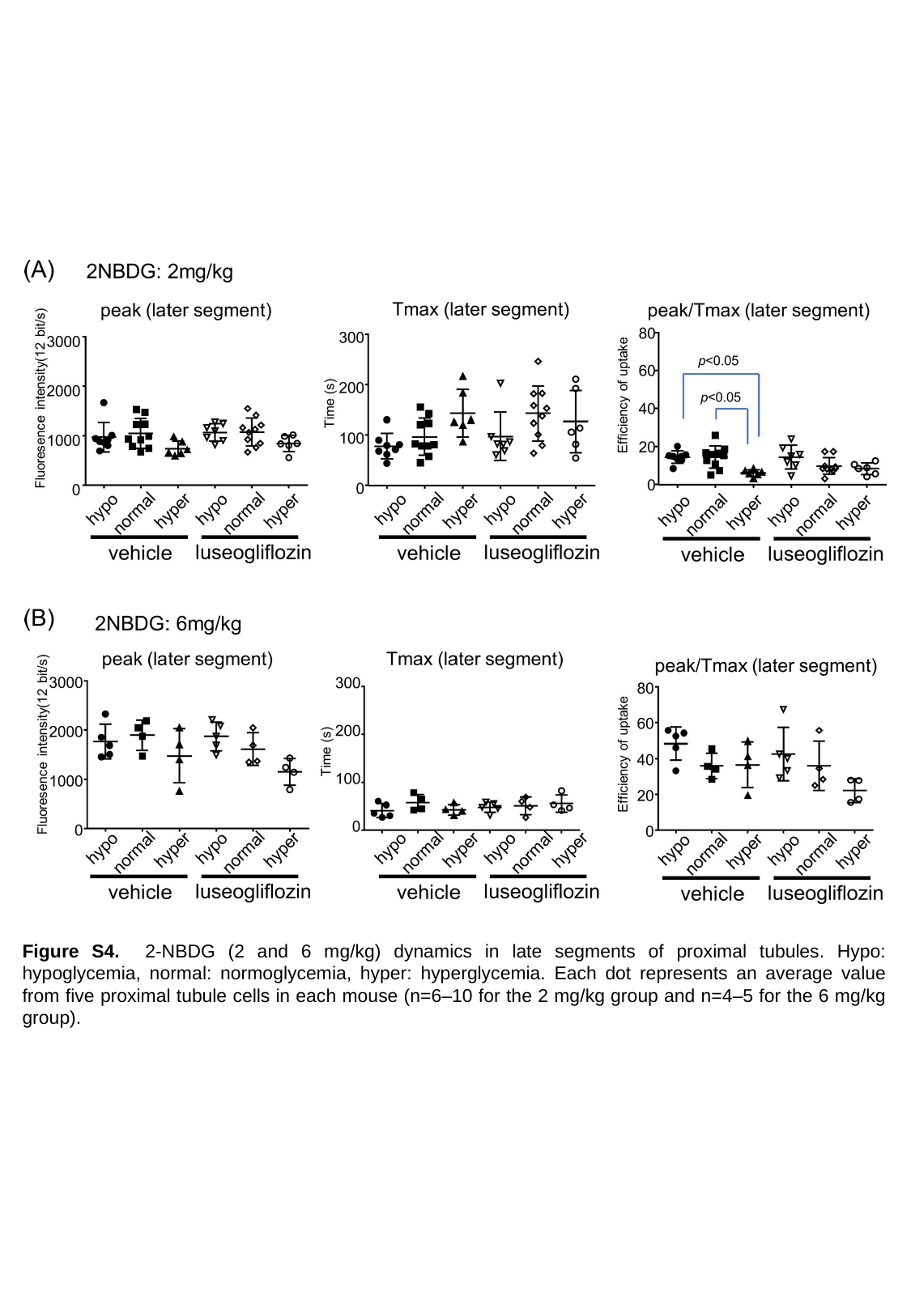

Figure S4. 2-NBDG (2 and 6 mg/kg) dynamics in late segments of proximal tubules. Hypo: hypoglycemia, normal: normoglycemia, hyper: hyperglycemia. Each dot represents an average value from five proximal tubule cells in each mouse (n=6–10 for the 2 mg/kg group and n=4–5 for the 6 mg/kg group).

## Slide 5
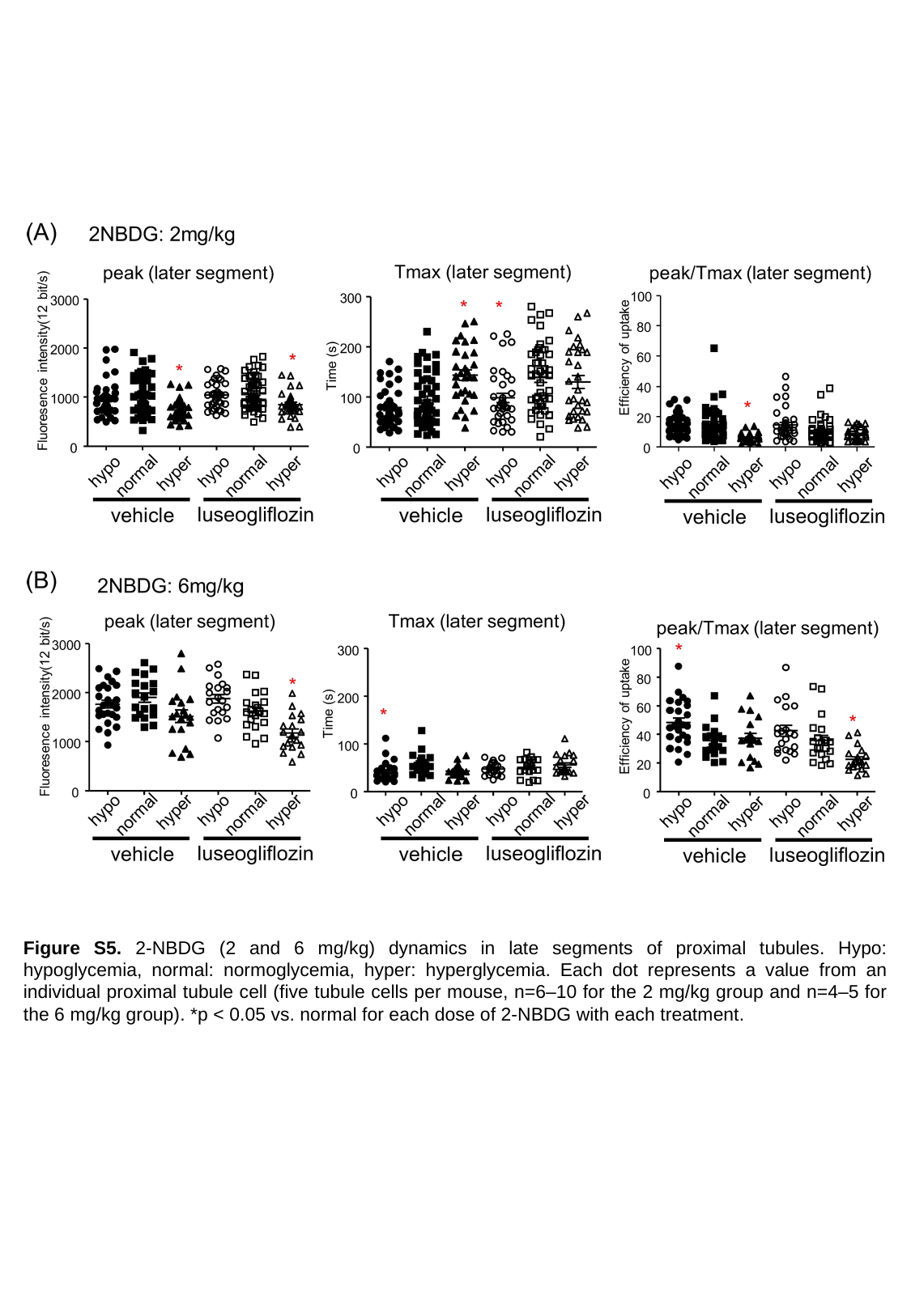

Figure S5. 2-NBDG (2 and 6 mg/kg) dynamics in late segments of proximal tubules. Hypo: hypoglycemia, normal: normoglycemia, hyper: hyperglycemia. Each dot represents a value from an individual proximal tubule cell (five tubule cells per mouse, n=6–10 for the 2 mg/kg group and n=4–5 for the 6 mg/kg group). *p < 0.05 vs. normal for each dose of 2-NBDG with each treatment.

## Slide 6
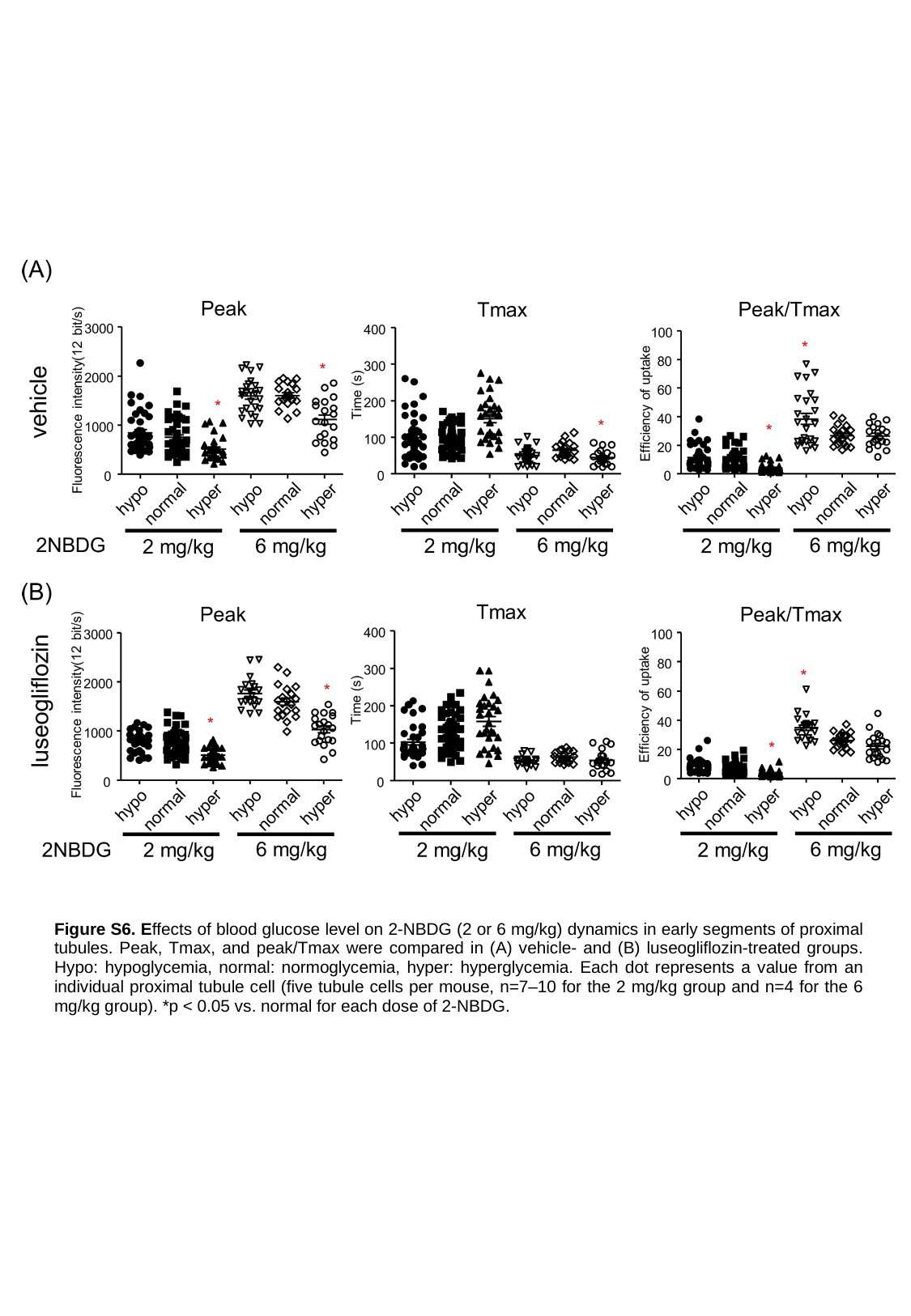

Figure S6. Effects of blood glucose level on 2-NBDG (2 or 6 mg/kg) dynamics in early segments of proximal tubules. Peak, Tmax, and peak/Tmax were compared in (A) vehicle- and (B) luseogliflozin-treated groups. Hypo: hypoglycemia, normal: normoglycemia, hyper: hyperglycemia. Each dot represents a value from an individual proximal tubule cell (five tubule cells per mouse, n=7–10 for the 2 mg/kg group and n=4 for the 6 mg/kg group). *p < 0.05 vs. normal for each dose of 2-NBDG.

## Slide 7
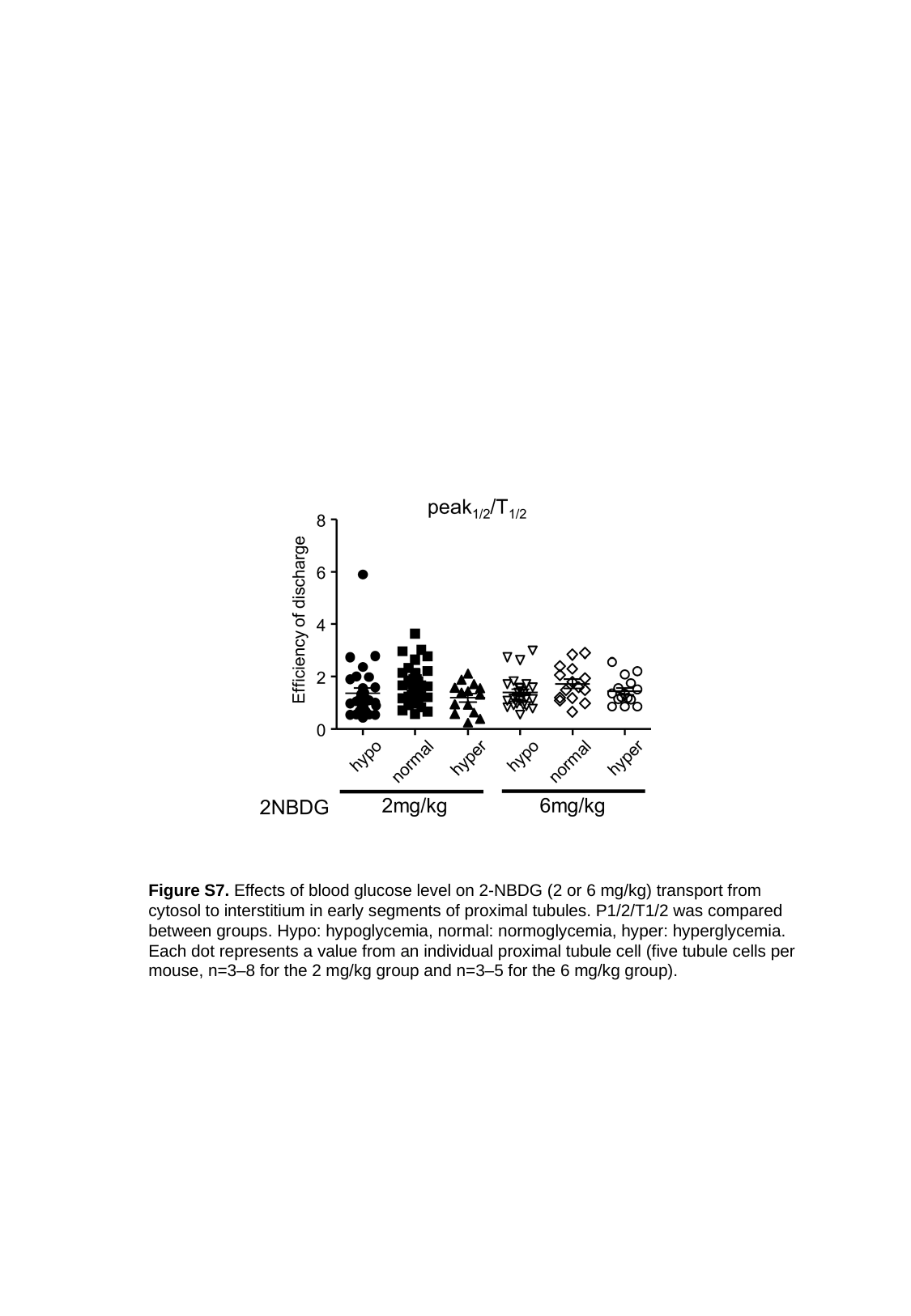

Figure S7. Effects of blood glucose level on 2-NBDG (2 or 6 mg/kg) transport from cytosol to interstitium in early segments of proximal tubules. P1/2/T1/2 was compared between groups. Hypo: hypoglycemia, normal: normoglycemia, hyper: hyperglycemia. Each dot represents a value from an individual proximal tubule cell (five tubule cells per mouse, n=3–8 for the 2 mg/kg group and n=3–5 for the 6 mg/kg group).

## Slide 8
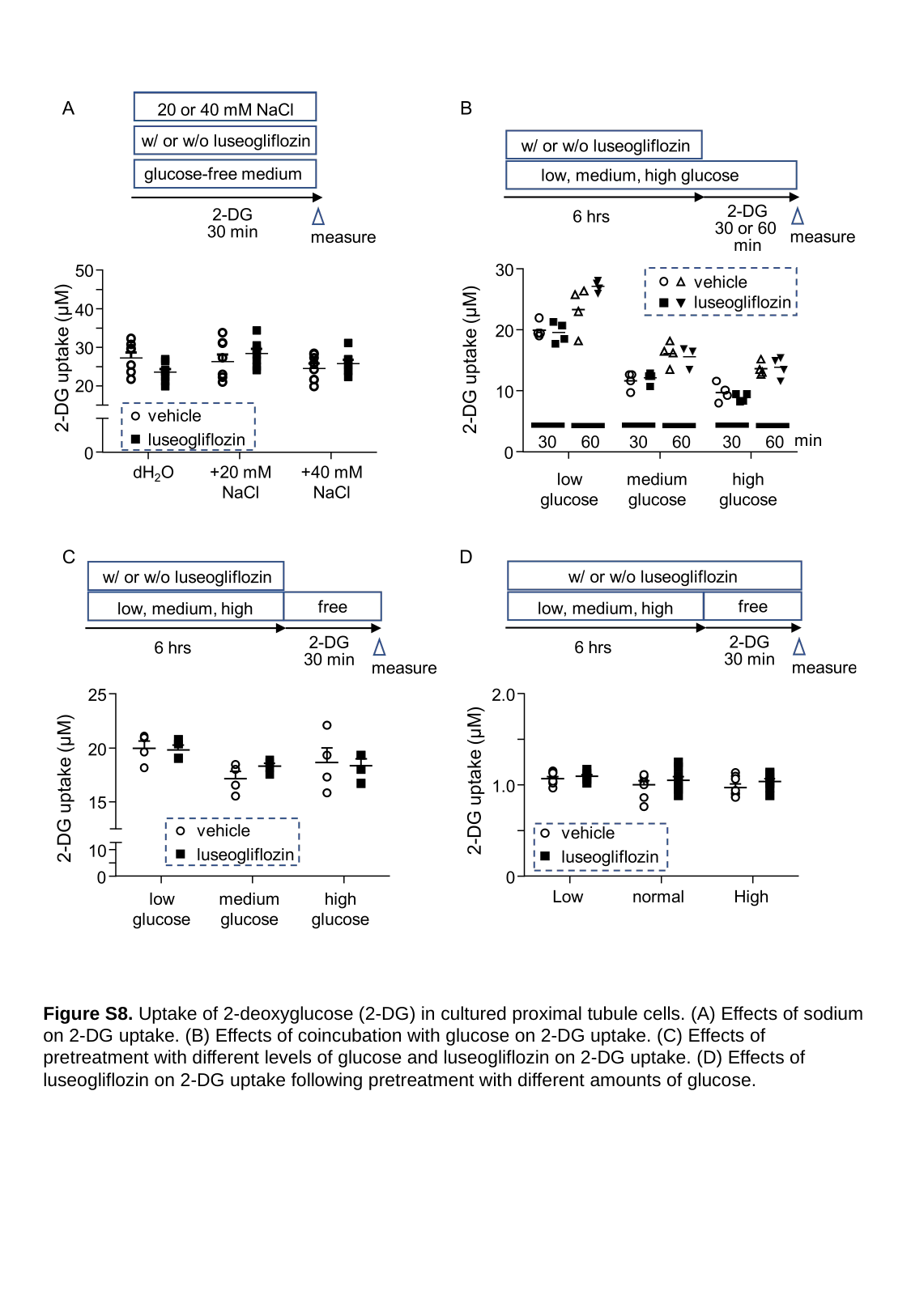

Figure S8. Uptake of 2-deoxyglucose (2-DG) in cultured proximal tubule cells. (A) Effects of sodium on 2-DG uptake. (B) Effects of coincubation with glucose on 2-DG uptake. (C) Effects of pretreatment with different levels of glucose and luseogliflozin on 2-DG uptake. (D) Effects of luseogliflozin on 2-DG uptake following pretreatment with different amounts of glucose.
